# Flapping Tremor: Unraveling Asterixis—A Narrative Review

**DOI:** 10.3390/medicina60030362

**Published:** 2024-02-21

**Authors:** Jamir Pitton Rissardo, Sara Muhammad, Venkatesh Yatakarla, Nilofar Murtaza Vora, Paras Paras, Ana Letícia Fornari Caprara

**Affiliations:** 1 Neurology Department, Cooper University Hospital, Camden, NJ 08103, USA; 2 Neurology Department, Mayo Clinic, Rochester, MN 55905, USA; muhammad.sara@mayo.edu; 3 Medicine Department, Terna Speciality Hospital, Navi Mumbai 400706, Maharashtra, India; yatakarlavenkatesh7@gmail.com (V.Y.); nilofar031202@gmail.com (N.M.V.); 4 Medicine Department, Government Medical College, Patiala 147001, Punjab, India; paras.jchander@gmail.com; 5 Medicine Department, Federal University of Santa Maria, Santa Maria 97105110, Brazil; ana.leticia.fornari@gmail.com

**Keywords:** asterixis, anisosterixis, mini-asterixis, myoclonus, hyperkinetic, encephalopathy, drug induced, movement disorder

## Abstract

Asterixis is a subtype of negative myoclonus characterized by brief, arrhythmic lapses of sustained posture due to involuntary pauses in muscle contraction. We performed a narrative review to characterize further asterixis regarding nomenclature, historical aspects, etiology, pathophysiology, classification, diagnosis, and treatment. Asterixis has been classically used as a synonym for negative myoclonus across the literature and in previous articles. However, it is important to distinguish asterixis from other subtypes of negative myoclonus, for example, epileptic negative myoclonus, because management could change. Asterixis is not specific to any pathophysiological process, but it is more commonly reported in hepatic encephalopathy, renal and respiratory failure, cerebrovascular diseases, as well as associated with drugs that could potentially lead to hyperammonemia, such as valproic acid, carbamazepine, and phenytoin. Asterixis is usually asymptomatic and not spontaneously reported by patients. This highlights the importance of actively searching for this sign in the physical exam of encephalopathic patients because it could indicate an underlying toxic or metabolic cause. Asterixis is usually reversible upon treatment of the underlying cause.

## 1. Introduction

Asterixis is a disorder of motor control, defined as sudden, brief, arrhythmic lapses of sustained posture due to involuntary interruption in muscle contraction [1,2]. This condition involves a specific form of negative myoclonus, characterized by momentary loss of muscle tone in agonist muscles, followed by a compensatory jerk involving antagonistic muscles [3]. Asterixis was originally described as a “liver flap” in the 1940s by liver specialists at the Thorndike Laboratory at Boston City Hospital, who noted the movement disorder in their patients [4]. It was also observed that this abnormal movement was common in metabolic encephalopathies and not only in patients with hepatic disease [5]. Reportedly, with the help of Father Cadigan, a Jesuit classic scholar from Boston College, Adams and Foley initially created a term to explain the asynchronous flapping: “anisosterixis”, from the ancient Greek, where an = negative, iso = equal, sterixis = solidity or firmness. Foley realized the word was too complicated to be used in clinical practice and simplified it to “asterixis” [4,5]. 

Asterixis is acknowledged as a significant albeit non-specific neurological manifestation linked with numerous conditions, with metabolic encephalopathies being the most prevalent culprit. It also has been reported in renal insufficiency and respiratory failure, both with hypercapnia and hypoxia, structural brain lesions due to cerebrovascular accidents, tumors, subdural hematoma, epidural abscess, polycythemia, septic encephalopathy, hyperviscosity, and medications [6,7].

It is possible to elicit asterixis against gravity, but when the wrist is in dorsiflexion, gravity accentuates the downward movement of the hand. There are early reports from Adams and Foley dating from 1953 describing asterixis also in proximal muscles of the upper limbs, lower limbs, neck, face, and tongue. It is also known that depending on the cause, asterixis could be fully reversible. Generally, when toxic-metabolic encephalopathy is treated and resolved, for example, so does the asterixis [7].

Asterixis is normally asymptomatic and not spontaneously noted by patients but is usually encountered during physical examination. In rare cases, asterixis can be a presenting feature reported by individuals as unusual jerky movements in the hands, problems with handwriting, or falls in the case of lower limb or truncal asterixis [2].

## 2. Etiology

Unilateral or bilateral asterixis can manifest, often with asynchronous, irregular, and varying frequency and amplitude. The clinical history of the possible underlying illness or toxic/metabolic process should be a starting point for further investigations, along with suggestive clinical signs, considering that asterixis is a highly non-specific sign associated with multiple causes. In 1973, Young and Shahani were the first to describe unilateral asterixis, and they classified involuntary movements as a form of “mini-asterixis” and the pauses as negative myoclonus [8].

Unilateral asterixis could arise from focal lesions in the thalamus, although cases of lesions in the midbrain, parietal cortex, and frontal cortex leading to unilateral asterixis have been reported [9]. In rare occasions, bilateral asterixis could be secondary to a unilateral lesion, for example, subdural hematoma causing mass effect [2]. 

A study of 45 cases with asterixis revealed ischemic and hemorrhagic disorders of the central nervous system (CNS) to be the most frequent causes of asterixis (95.5%), and the thalamus was the most frequent localization for unilateral asterixis (54%) [10]. Bilateral asterixis is commonly linked to metabolic encephalopathies, particularly of hepatic origin. Patients with cardiac and respiratory failure, uremia, electrolyte imbalances (primarily hypoglycemia, hypokalemia, and hypomagnesemia), and drug intoxication may also exhibit bilateral asterixis. An array of drugs can induce asterixis, with phenytoin intoxication being the most frequently reported, followed by benzodiazepines, barbiturates, valproate, gabapentin, carbamazepine, lithium, ceftazidime, and metoclopramide [11].

In terms of other generalized encephalopathies, asterixis has also been associated with cerebral malaria, probably due to impaired microvascular circulation [12], Creutzfeldt–Jacob disease, where it could be secondary to non-inflammatory changes associated with malformed proteins and encephalitides to brain inflammation caused by infection [13] and viral encephalitis probably in the context of neuroinflammation [14]. Cerebral trypanosomiasis has also been associated with bilateral asterixis in a case report. In the latter, treatment with eflornithine led to the resolution of obtundation and asterixis and considerable resolution of brain magnetic resonance imaging (MRI) abnormalities. In this way, the early consideration of non-CNS sources of infection in patients initially presenting with encephalopathy and asterixis is important. Epidemiological factors such as travel history, occupation, sexual contacts, and vaccination history should be taken into account to guide microbiological investigations [7].

Asterixis is more often noticed in the upper extremities than in the lower. Nevertheless, there have been reports of asterixis that are more noticeable on the lower extremities than the upper. This particular presentation could be explained by altered mental status and the patients’ difficulties in obeying commands and performing dorsiflexion of the wrists. However, the predominance of asterixis in lower extremities has also been reported in alert patients. It is also commonly seen that as asterixis worsens in the upper extremities, it gradually becomes more prominent in the lower extremities [7].

In a prospective study in the inpatient and outpatient Neurology services, it was noticed that in individuals with worsening renal dysfunction, blood urea nitrogen, and creatinine, the severity of the asterixis worsened. However, in patients with hepatic disease, liver markers (aspartate transaminase, alanine transaminase, and alkaline phosphatase), as well as ammonia levels, did not correlate directly with the severity of asterixis [7].

## 3. Pathophysiology

**Figure 1:**
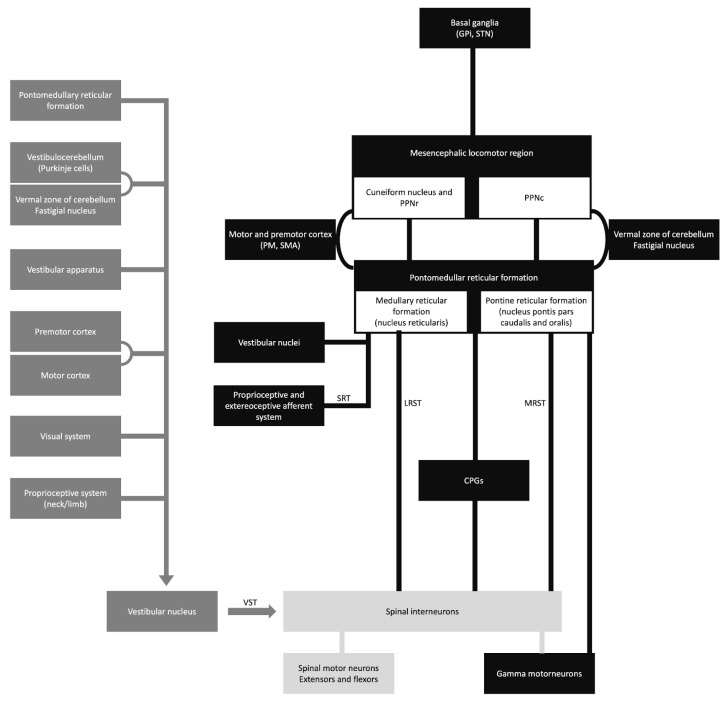
Schematic diagram of the sensory-motor control. CPGs, central pattern generators; GPi, globus pallidus internus; LRST, lateral (medullary) reticulospinal tract; MRST, medial (pontine) reticulospinal tract; PM, premotor cortex; PPNc, caudal region of pedunculopontine nucleus; PPNr, rostral region of pedunculopontine nucleus; SMA, supplementary motor area; SRT, spinoreticular tract; STN, subthalamic nucleus; and VST, vestibulospinal tract.

The vestibulospinal, reticulospinal, and rubrospinal tracts are the main tracts related to the postural stability or tonic control of the extremities. The modulation of these tract functions is controlled by supratentorial structures (Figure 1) [15]. In this context, the cerebello-rubral and vestibulocerebellar fibers converge to the ventrolateral nucleus of the thalamus and some to the prefrontal cortical area [15]. Another interesting fact is that some muscle tone and postural regulation occur by the medial frontal cortex, which projects to the brainstem reticular formation [15]. The transient motor symptoms and the common bilateral presentation suggest that the postural tone control is mainly bilateral.

Asterixis, initially observed by Adams and Foley in 1949, is usually a bilateral tremor characterized by flapping movements. It is often considered an indicator of subcortical negative myoclonus, a rhythmic motor phenomenon [16]. The presence of this condition has been reported in various metabolic encephalopathies as an adverse effect of drugs and structural abnormalities. Unilateral asterixis has been reported in structural brain lesions [10].

The pathophysiology of asterixis is still unknown. It was hypothesized as a disturbance of the ascending activating systems, affected by conditions like encephalopathy and related to lesions in the thalamus and midbrain [17]. Possible brain areas that can explain the pathophysiology of asterixis are the parietal lobe and midbrain. These cortical regions are mainly related to sensorimotor integration, and their dysfunction can lead to the receptive inattentiveness of incoming information [17].

Electrophysiological assessment has identified the presence of negative sharp waves in the opposite central area, indicating abnormal motor activity in the cortex [18]. The origin of mini-asterixis is thought to arise from the involvement of the motor cortex, which results in motor cortical waves being slowed down and synchronized [19]. Moreover, patients with cerebellar lesions can present with ipsilateral asterixis, which can be explained by the crossing of the cerebello-rubral fibers at the superior cerebellar peduncle.

## 4. Diagnosis

### 4.1. Clinical Assessment

**Figure 2:**
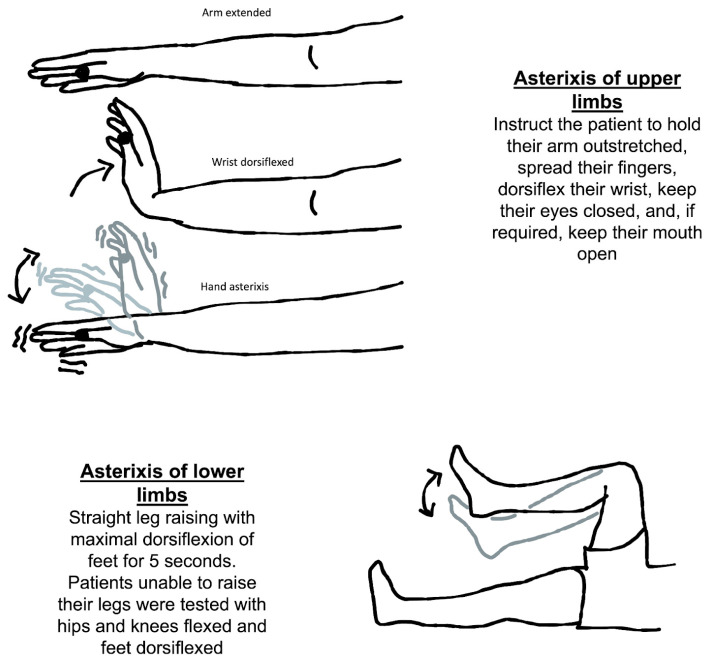
Neurological assessment of asterixis of the upper and lower limbs.

The usual method for eliciting asterixis is to instruct the patient to hold their arm outstretched, spread their fingers, dorsiflex their wrist, keep their eyes closed, and, if required, keep their mouth open (Figure 2). Then, looking for any abnormally brief downward flaps of the hands that return quickly to their previous posture is possible. If it is not obvious immediately, the patient can be instructed to keep their arms straight while the examiner softly extends their wrist in a sweeping motion. There is a significant latent period between adopting the posture and the beginning of the asterixis, so it is important to wait at least thirty seconds before concluding that the sign is absent. Also, the examiner should not mistake asterixis for clonus, which is a rhythmic involuntary muscular contraction induced by the sudden passive stretching of a muscle or tendon. Clonus is a rhythmic oscillating stretch reflex response that involves rhythmic movement. Additionaly, clonus is generally accompanied by hyperreflexia and could be seen in upper motor neuron lesions. It could be elicited in the ankle by briskly flexing the foot of the patient.

Another way to check for asterixis is to have the patient lie on their back on the bed with both their knees bent for evaluation of symmetry. The patient should be told to let their legs down. When the legs fall to the sides, the feet should be flat on the table. It is important to observe any flapping of the legs at the hip joint. The knees repeatedly come back together as a result of this. It has also been discovered that asterixis cannot be properly induced without evaluating the limbs against gravity [20].

There are some alternative approaches to evaluating asterixis (Table 1). The examiner can advise the patient to squeeze his hand or extend his fingers. Patients who struggle with posture are frequently unable to squeeze steadily [3]. Another possible way to evaluate asterixis in patients is to instruct the patient to squeeze a blood pressure cuff that is only partially inflated while being told to keep the reading steady. In patients with asterixis, the values jump wildly [3].

The severity of asterixis can be determined according to the joints being affected and the distribution (Table 2).

### 4.2. Asterixis in Any Skeletal Muscle

Adams and Foley described that asterixis can occur in any skeletal muscle in which voluntary musculature is required to maintain posture [21]. The most common location of asterixis is the wrist, but severe cases can reveal the presence of this phenomenology in the tongue, lips, and eyelids [7]. Interestingly, asterixis usually occurs asynchronously on either side of the body, except when it involves the facial muscles [21]. The inspection of the tongue should be done as it rests inside the mouth. Afterwards, the protrusion can be examined.

One location of asterixis that has still not been reported is the extraocular muscles. The clinical detail of the movement and its amplitude were probably some factors associated with the scarce description in the literature. Another hypothesis could be that asterixis simply does not occur in extraocular muscles.

### 4.3. Diagnostic Assessment

Complete blood cell count, electrolytes, glucose, renal function tests, liver function tests, and arterial blood gas analysis are the recommended laboratory studies to rule out metabolic conditions associated with asterixis. In individuals using medications, if a patient’s history points to drug intoxication, it is important to investigate drug-induced asterixis. Neuroimaging can help locate possible lesions in the CNS associated with asterixis. In the right circumstances, it is also possible to examine non-vascular causes, including infections and malignancies [3].

The mean frequency of movements has been historically reported to be around 3–5 Hz. However, in clinical practice, it is usually less, around the range of 0.5 to 2 Hz. Very fine asterixis affecting the fingers can be easily confounded with tremors [2]. The main differences between asterixis and tremor will reside in the phenomenology of the movement. Asterixis is usually antigravitational, but, in some cases, the differentiation can only be appreciated with electrodiagnostic studies. The cerebral origin of mini-asterixis in hepatic encephalopathy was assessed by Timmerman et al. by examining hand muscle electromyographic (EMG) recordings and brain activity captured by magnetoencephalography, and it was highlighted that this technique might be utilized to distinguish between different tremor disorders [22].

EMG features include a 35 to 200-millisecond electrical activity pause in many muscles. For example, the affected limb may jolt back into place or be impacted by gravitational or tendinous elastic stresses. This is followed by sudden motor unit activation [3]. In this context, the silent period locked averaging method was described by Ugawa et al. and uses a backward averaging methodology to analyze asterixis [23]. This approach can be used to investigate the causes of asterixis as well as the many types of EMG silences that accompany it.

Asterixis involving the facial muscles and the tongue can easily be misdiagnosed as dyskinesia. Some facts can help to differentiate these two clinical manifestations. Firstly, dyskinesia usually involves more than one muscle, so the presentation frequently involves the tongue and other orofacial muscles. Secondly, these two movement disorders have different phenomenology. Dyskinesia occurs throughout the movement, and asterixis is posture related [24].

The clinical significance of asterixis is that the presence of this movement disorder suggests some degree of neurological involvement and could potentially indicate the presence of a severe underlying toxic or metabolic process, even though the occurrence of disorder in itself is insufficient to provide a differential diagnosis. Interestingly, Hardison et al. used only asterixis as a sign to admit patients with alcohol-associated liver disease, and they observed that this physical finding had prognostic value and predicted mortality. Approximately 56% of the individuals with asterixis had a poor outcome [25]. In another study, patients with asterixis were older, had worse liver function, and worse neuropsychiatric performance compared to a group of individuals without the sign. Moreover, patients presenting with asterixis at admission were more likely to have unplanned readmission within a thirty-day period [26].

## 5. Differential Diagnosis

Asterixis has been extensively studied in adults, with an estimated incidence varying from 0.47 to 8.75% of neurological consultations [7,27]. The differences between the percentages can be explained by two factors. First, patients usually do not spontaneously report asterixis. Second, some of the studies performed detailed examinations of all the patients where the investigator actively searched for asterixis in the physical exam.

Interestingly, the literature regarding asterixis in the pediatric population is scarce. Aravamuthan et al. found only 0.06% of the pediatric individuals presented with asterixis in a tertiary care pediatric hospital [28]. All the cases of pediatric asterixis were side effects of medications, and the neurologists were consulted for complaints unrelated to asterixis, such as requested a second opinion regarding non-improvement in seizure attacks after optimal therapy [28].

The presence of bilateral asterixis at presentation suggests metabolic abnormalities and side effects of medications. The metabolic conditions associated include but are not limited to hepatic encephalopathy, renal failure/azotemia, respiratory failure, electrolyte disturbance, heart failure, Wilson’s disease, and hypoglycemia. In the cases of drug-induced asterixis, there are reports of individuals using alcohol, barbiturates, carbamazepine, antipsychotics (clozapine, lithium), phenytoin, gabapentin, metoclopramide, and levodopa.

It is worth mentioning that bilateral asterixis can present with unilateral structural brain lesions (Table 3). These presentations were rarely reported in the literature and can be challenging to diagnose.

**Figure 3:**
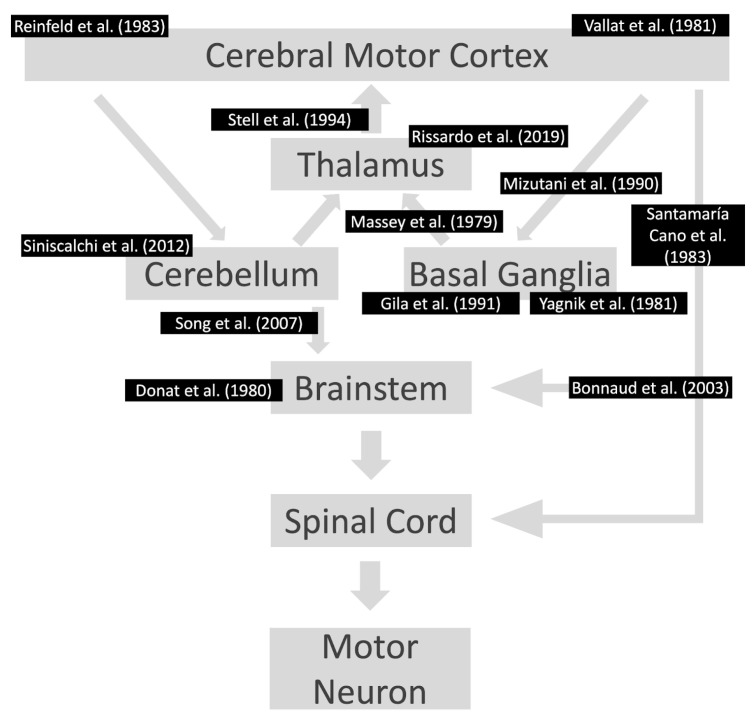
Lesions already reported in the motor pathway associated with asterixis. The references are according to the area affected in the motor pathway. Notably, the individuals had contralateral motor symptoms at the location of the lesions. References: [20,31,39,41,42,43,44,45,46,48,50,51,53].

In cases of unilateral presentation of negative myoclonus, focal structural brain lesions in the genu and the anterior portion of the internal capsule or ventrolateral thalamus should be investigated (Figure 3) (Table 4) [7,20,31,39,40,41,42,43,44,45,46,47,48,49,50,51,52,53]. In this context, cerebellar lesions sometimes cause ipsilateral asterixis, which can be explained by the decussation of dentato-rubro-thalamo-cortical fibers before they pass through or create synapses with the red nucleus. In this way, it is imperative to conduct a complete neurological examination in these patients because accurate diagnosis frequently relies on careful attention to neurological signs and symptoms. There is a broad differential diagnosis for cerebellar pathologies, and alterations in coordination, eye movements, and balance could indicate an underlying vascular lesion for example which should be promptly managed [54]. No unilateral asterixis reports associated with contralateral brain lesions have been reported, with good anatomy documentation (imaging or autopsy) and case-by-case documentation of normal blood chemistry.

One crucial differential diagnosis of asterixis is pseudoasterixis. Subtle movements may trigger pseudo-asterixis so that it can simulate asterixis. Pseudoasterixis is defined as brief, rapid, voluntary action tremors of the hands and fingers, elicited by slow flexion and extension movements of the hands at the wrists while keeping the fingers in full hyperextension. Another fact to differentiate asterixis from pseudo-asterixis is that the patient is aware of the hand twitching when tested in pseudoasterixis [55]. Noteworthy is that pseudoasterixis is rarely observed in clinical practice [7].

### 5.1. The Liver Flap (Asterixis) in Hepatic Encephalopathy

**Figure 4:**
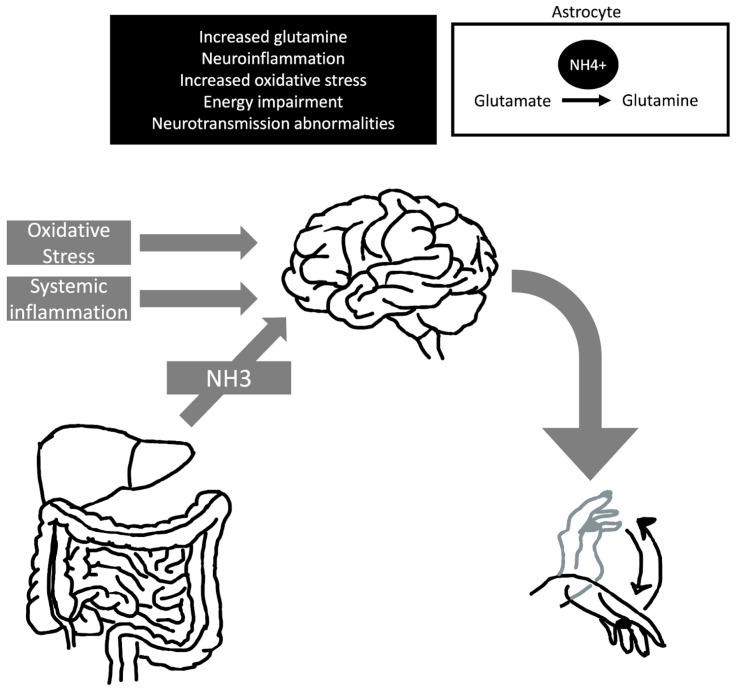
Pathophysiology of hepatic encephalopathy and asterixis. The development of asterixis in hepatic encephalopathic individuals is probably multifactorial, and it can associate with hyperexcitability of motor cortical areas. Abbreviations: NH3, ammonia; NH4+, ammonium cation.

Asterixis is a common finding in hepatic encephalopathy (Figure 4). It can be assessed by having the patient hold their arms in a fixed dorsiflexion position, which reveals the inability to maintain a posture. It has also been reported with tongue protrusion or dorsiflexion of the foot [19]. Asterixis does not occur in early or advanced hepatic encephalopathy. Asterixis tends to disappear in correlation with worsening hepatic encephalopathy as the patient becomes comatose. As the condition improves, the patient often recovers without long-term neurological impairment [56].

The West Haven criteria is one of the most frequently used instruments for grading hepatic encephalopathy (Table 5). The categories in HE can be graded from I to IV and are based on various clinical parameters and the presence or absence of asterixis [57]. In grade I, patients have inattention and personality changes, mild incoordination, and difficulty with handwriting. Grade II is characterized by asterixis, ataxia, paratonia, dysarthria, apraxia, disorientation, lethargy, and inappropriate behavior. In grade III, patients may present hyperreflexia, Babinski’s sign, and spasticity, and they usually respond to stimuli but are stuporous. Grade IV is characterized by thalamic–subcortical spasticity of the entire body and coma [58]. It is noteworthy that evaluating asterixis and the neuromuscular state may be the most objective part of the physical exam of patients with HE. The assessment of this condition is usually challenging given the subjective nature of other parameters, such as mental health status and behavioral changes.

The current guidelines to treat asterixis recommend lactose enemas or neomycin tablets; both treatments have comparable effects on improving asterixis [60]. A systematic review found that rifaximin is equally effective for improving mental status and lowering asterixis as nonabsorbable disaccharides and other drugs, but it has a better safety profile [61].

### 5.2. Hyperammonemia and Asterixis

The first neuropsychiatric symptoms associated with ammonia occur at concentrations above 60 µmol/L, which include appetite loss, nausea, insomnia, agitation, and personality changes [62]. Singh et al. reported a patient who developed asterixis following carbamazepine use that showed an isolated increase in ammonia without an increase in liver enzymes, suggesting hepatocellular dysfunction without cellular damage or isolated mitochondrial dysfunction [63]. One of the possible therapeutical choices for managing ammonia levels is the prescription of L-carnitine, which has been used in cases with urea cycle disorders and valproic-acid-related toxicities to reduce serum ammonia levels [64].

Asterixis has also already been reported with carnitine deficiency. Carnitine is an essential co-factor implied in fatty acid metabolism, and its deficiency can impair fatty acid oxidation, leading to hyperammonemia and encephalopathy in rare cases. Limketkai et al. reported the case of a 35-year-old woman who developed acute mental status changes, asterixis, diffuse muscle weakness, and hyperammonemia at 276 μg/dL [65].

Interestingly, asterixis has also been reported in the context of valproate-induced hyperammonemia encephalopathy (VHE), which is a rare but serious adverse effect of valproic acid characterized by varying degrees of altered mental status, vomiting, and focal neurological deficits [66]. In psychiatric patients, for example, the concomitant use of other medications could precipitate a rise in serum ammonia levels [67]. In a study by Chopra et al., among psychiatric patients, 51% (*n* = 123) of patients receiving valproic acid had asymptomatic hyperammonemia (level > 97 μg/mL). The symptoms of VHE can occur days or years after the initiation of valproic acid regardless of normal therapeutic doses and serum valproic acid concentrations [68].

In a prospective study investigating portosystemic encephalopathy (PSE), 30 patients were evaluated after undergoing transjugular intrahepatic portosystemic shunts. Interestingly, the changes in the asterixis grade mirrored the changes in mental status. There was significant worsening in the first month after the transhepatic intrajugular portosystemic shunt (TIPS) and then a return to baseline by six months. On the other hand, there were no significant changes in serum ammonia levels compared to either baseline or controls. However, there were trends toward increased plasma ammonia levels over time in patients who underwent TIPS. The authors suggested that portosystemic encephalopathy may be directly related to “sinusoidal steal” plus hepatocyte dysfunction after a portosystemic shunt. They also mentioned that after TIPS, a further decrease in sinusoidal flow may cause more pronounced effects on hepatocellular function and increase the likelihood of PSE in patients with the most severe abnormalities in sinusoidal perfusion before TIPS, thereby worsening PSE. Additionally, we hypothesize that with TIPS, there might be a transient and sudden increase in blood ammonia levels, which could be hard to detect in laboratory analysis but could contribute to the onset of acute encephalopathy and the occurrence of asterixis [69].

### 5.3. Drug-Induced Asterixis

Asterixis was reported as a complication of clozapine, especially when taken with other antipsychotic medications such as lithium and carbamazepine. In a study of 10 patients treated with clozapine (eight patients), lithium (seven patients), and carbamazepine (seven patients), there were neither metabolic disorders nor structural brain lesions that could explain the occurrence of asterixis [70]. A similar case of asterixis was reported with the combination of lithium, clozapine, and zuclopenthixol [71].

Friedman et al. studied a cohort of patients with clozapine whose asterixis was investigated, and the authors found two main features. First, asterixis was observed, but the patients were asymptomatic, and some reported falls rarely. Second, there was no dose-dependent side effect, and asterixis was observed even with low doses of clozapine, suggesting a threshold effect [72].

The occurrence of asterixis can be attributed to the direct effect of the drug on the CNS and the indirect effect (Table 6) [7,70,73,74,75,76,77,78,79,80,81,82,83,84,85,86,87,88,89,90,91,92,93,94,95]. The direct effect can be explained by abnormalities in the neurotransmitter levels and toxic levels of the medications. On the other hand, the indirect effect is usually observed with medications that lead to hepatic dysfunction and increased levels of ammonia, predisposing the individual to develop asterixis.

Some medications should be cautiously prescribed due to their narrow therapeutic index and propensity to toxic levels. In individuals with chronic renal failure, gabapentin dosage should be carefully increased. There are reports of gabapentin intoxication presenting with asterixis before other neurological symptoms [82]. Lithium [89] and amantadine [79] are other medications associated with asterixis. Interestingly, in the reports with lithium, triphasic waves were already noticed [89]. However, asterixis associated with lithium has already been reported at therapeutic and toxic levels [96].

Identifying asterixis in its initial stages can enable the initiation of prompt and efficient treatment, potentially preventing complications in these patients [3]. However, most individuals will complain of unspecific clinical manifestations before the development of asterixis, and the presence of asterixis already represents a significant involvement of the CNS. Thus, it is essential to closely monitor patients taking a combination of psychotropic medications [11].

### 5.4. Epileptic Negative Myoclonus

Myoclonus is defined as a sudden, brief, jerky, shock-like, and usually irregular, involuntary movement. When secondary to a muscular contraction, it is denominated positive myoclonus, and when caused by an interruption of muscular activity, negative myoclonus. Electrophysiologically, negative myoclonus is also characterized by a sudden cessation of muscle contraction or a silent period of EMG discharge without accompanying contraction of the antagonist’s muscles [97].

The definition of negative myoclonus was coined in 1976 by Shahani and Young to describe the myoclonic-like movement associated with the brief pauses in the ongoing voluntary muscle activity that characterize post-hypoxic intention myoclonus and asterixis. The current definition of negative myoclonus also includes any brief, jerky interruption of tonic muscle activity that causes a sudden postural lapse. The International League Against Epilepsy recently recognized “negative myoclonus” as a type of seizure characterized by “interruption of tonic muscle activity for <500 milliseconds without evidence of preceding myoclonia [98]”.

Although classically negative myoclonus has been used as a synonym of asterixis, Obeso et al. proposed a distinction of negative myoclonus in four subtypes [99]. The first is physiological negative myoclonus, detectable in normal subjects when falling asleep, for example, or provoked by unexpected and sudden stimuli. Asterixis is the second category, followed by the third category, which is postural lapses, characterized by abrupt interruptions of tonic muscular activity of postural muscles ranging from 200–500 milliseconds, as in post-hypoxic action myoclonus; and finally, epileptic negative myoclonus [97].

Considering most myoclonus types are associated with enhancement of the neuronal activities that are present in healthy individuals, it might be useful to adopt this classification to increase diagnostic accuracy. Moreover, further electrophysiological studies should be performed to elucidate the pathophysiological mechanism of the underlying disorder [97].

Epileptic negative myoclonus (ENM) is a non-specific motor disturbance that can depend on dysfunction at different anatomo-functional levels, including premotor and motor cortex [100]. It manifests across a spectrum of epileptic conditions, from discrete alterations like benign epilepsy with centrotemporal spikes (BECTS) to severe epileptic encephalopathies. Clinically, ENM can present as a very mild motor event. On the other hand, it can also cause patients to drop objects and report “tremulousness” of a limb during daily activities, head nodding, sudden falls, and even fecal incontinence when pelvic floor muscles are involved [97]. Neurophysiological studies indicate a cortical origin of ENM, supported by EEG mapping and spike analysis, particularly involving centroparietal and frontal motor areas. The onset and duration variability of ENM suggest involvement not only from cortical but also from subcortical and pontine structures [100].

Studies have shown that ethosuximide is effective in treating ENM in children. Evidence indicates that this medication, by acting as a blocker of T-type calcium channels in thalamic neurons and associated cortical networks, might regulate the underlying mechanisms of ENM at the thalamocortical level. Levetiracetam has also been reported to be beneficial in ENM in preliminary findings. However, more studies are needed to confirm the effectiveness of this drug. It has also been described that ENM can be worsened, especially in children with focal epilepsies, by the use of agents such as carbamazepine, valproic acid, phenytoin, lamotrigine, and oxcarbazepine [98].

### 5.5. The Uremic Flap-Asterixis in Renal Failure

The most common movement disorders in individuals with chronic kidney disease are restless legs syndrome, myoclonus, and asterixis [101]. Even though being frequently found in chronic kidney disease, asterixis usually does not require treatment because it does not significantly impair the quality of life of the patients.

Uremic encephalopathy is a spectrum of metabolic abnormalities that range from inattention to coma. It is reported that the accumulation of uremic toxins in renal failure leads to an imbalance between excitatory and inhibitory neurotransmitters [102]. Uremic encephalopathy presents as alterations in mental function and/or motor coordination. Its severe manifestation poses a risk for both morbidity and mortality. Mental alterations present as memory deficits, depression, significant cognitive impairments, and, in the most extreme cases, a widespread disorder marked by confusion, delirium, psychosis, seizures, coma, and ultimately death [103].

Uremic encephalopathy can present with intermittent loss of muscular tone in an outstretched arm, known as asterixis. Advanced uremic encephalopathy is characterized by a reduced level of awareness, anorexia, and upper motor neuron signs that might lead to ataxia and abnormalities in speech [103]. The diagnosis of uremic encephalopathy is mainly dependent on clinical presentation. The EEG can be abnormal with generalized slowing of delta waves, but usually nonspecific [104]. Most uremia-related CNS abnormalities can be reversed with dialysis in days or weeks, though mild symptoms might persist. Adjusting dialysis doses can aid patients with persistent symptoms. Successful renal transplantation often resolves uremic encephalopathy within days.

Some authors included the presence of asterixis in the diagnosis criteria of dialysis disequilibrium syndrome. Asterixis was classified as a moderate symptom of this syndrome. Port et al. suggested that dialysis disequilibrium syndrome should be diagnosed if there was onset of one severe (psychosis, convulsion, stupor, or coma), two major (asterixis, myoclonus, somnolence, or disorientation), or three minor (headache, vomiting, drowsiness, restlessness, or muscle cramps) symptoms [105].

### 5.6. Asterixis in Stroke and Transient Ischemic Attacks

Bilateral and unilateral asterixis may be considered a focal neurological sign in specific situations. This is a short-lasting sign in patients with focal brain lesions and occasionally may present in the setting of transient ischemic attacks. Although not definitely localizing, a unilateral asterixis is indicative, in most instances, of a contralateral hemispheric lesion, especially at the thalamic area, and often, a cerebrovascular accident is an underlying cause. In this way, carefully examining patients and noticing the presence of unilateral asterixis is essential because the laterality may help guide the diagnostic rationale. Only a few patients with non-stroke etiology manifested this focal sign. This may indicate that contrary to bilateral asterixis resulting from slowly evolving metabolic disturbances, lateralized asterixis often results from an acute disruption of neuronal circuits, and chronic focal lesions rarely produce this sign. This susceptibility to acute injury and the transient nature of this sign may be explained by a wide variety of neural pathways affecting postural control that leads to rapid neuronal adjustment and recovery. However, a pathophysiological explanation for this phenomenon has yet to be described.

Unilateral asterixis has been found in many cases of stroke, mainly in locations including the cerebellum [53], posterior thalamic-subthalamic paramedian region [106], midbrain [107], and pons [108]. Therefore, brain MRI and magnetic resonance angiography should be performed to evaluate unilateral asterixis further.

Unilateral asterixis is seen with a prevalence of 1.9% in patients with focal post-stroke brain lesions [109]. Asterixis secondary to stroke is usually moderate and associated with other manifestations, such as motor or sensory deficits and cerebellar syndrome. It is also observed after the disappearance of these symptoms when it must then be differentiated from a proprioceptive deficit [110]. Asterixis associated with stroke usually occurs in the acute phase of stroke, and the main involved circuitry is the dentato-rubro-thalamic system.

Pitton Rissardo et al. studied cases of limb-shaking associated with transient ischemic attack in the literature [111]. They found that asterixis is the second most common description of limb-shaking, followed by limb-shaking itself. Interestingly, unspecific jerkings and myoclonus were more commonly reported than asterixis [111]. In this context, the most common etiology for these patients presenting with asterixis and diagnosed with limb-shaking transient ischemic attack is clinically significant stenosis of the carotid arteries [112]. However, the diagnosis of a limb-shaking transient ischemic attack should be made after other causes are ruled out. Hanazono reported a case of a patient misdiagnosed with asterixis of limb-shaking TIA, which upon further investigation revealed callosal infarction secondary to severe anemia associated with colon cancer. This case is interesting because the patient’s asterixis immediately improved after blood transfusion, suggesting a relationship between anemia and neurological symptoms [113].

In a case series, Sayadnasiri et al. reported three patients with stroke that presented with unilateral or asymmetrical asterixis. The first patient had left hemiparesis and asterixis that were secondary to a right thalamic hemorrhage. The second patient presented with right-sided hemiparesis and asterixis after acute left thalamic infarction. The third individual had right-sided weakness and bilateral asymmetrical asterixis, which later subsided and was probably due to a transient ischemic attack localizing to the left internal carotid territory [114].

### 5.7. Asterixis in Respiratory Failure

In one of the first case series of asterixis associated with respiratory failure, Austen et al. described four patients who were “drowsy and inattentive (…)” and had “tremor and twitching of the extremities (…)” associated with hypercapnia and hypoxia. They described the tremor as visible only in outstretched fingers, and present during contraction of the forearm muscles and hands. The coarse twitching was seen when muscles were activated and maintained in a state of contraction and was described as arhythmic and asynchronous, identical to the “asterixis” seen in hepatic coma [115].

Asterixis may be associated with either hypercapnia or hypoxia and, therefore, is not a pathognomonic sign of type 2 respiratory failure, as occasionally suggested. In fact, asterixis has already been reported in a case series in patients with hypoxia without hypercarbia [7]. Michaelides et al. studied asterixis and mental confusion in individuals with hypercapnic chronic obstructive pulmonary disease patients. They observed in their cohort that the patients presenting with asterixis usually had elevated ammonium levels of 76.5 ± 15.7 μg/dL. Also, the presence of cor pulmonale was a determinant factor for the occurrence of asterixis during the follow-up. The authors proposed that the elevated blood levels of ammonia occurred due to liver dysfunction in the setting of cor pulmonale [116].

Conn et al. reported a patient with Cheyne–Stokes respiration who presented with altered mental status and asterixis more marked at the end of each apneic period. The author suggested that these fluctuations were secondary to anoxia since asterixis may disappear after oxygen administration. He hypothesized that the depression of the reticular formation of the CNS could be responsible for the delirium seen in these patients since the ascending reticular formation plays a role in wakefulness, attention, and integration of sensory impulses. He also mentioned that the descending reticular system is involved in maintaining muscle tone and posture and in the coordination of motor activity, which could explain the pathophysiology of asterixis in the context of respiratory failure [117].

Interestingly, Kim et al. reported a case of chronic negative myoclonus diagnosed in a 66-year-old man with COPD. The patient had been healthy before the sudden development of myoclonus in the forearm and trunk four months earlier. This sign was evident at rest and pronounced with an outstretched hand posture. No significant medical history or toxic exposure occurred. Complete evaluation, including brain MRI, CBC, and thyroid function tests, were normal, except for the chest X-ray, which showed emphysematous changes and arterial blood gas, which showed hypoxia and hypercapnia (pH 7.399, pCO_2_ 53.2 mmHg, pO_2_ 45.1 mmHg, HCO_3_ 32.2 mmol/L, O_2_ saturation 82.5%). Additionally, echocardiography revealed moderate pulmonary hypertension and chest computed tomography displayed bronchiectasis combined with emphysema. Electroencephalogram (EEG) showed intermittent sharp waves from the right frontal area and generalized slow waves, suggesting features of focal seizures arising from the right frontal area and mild cerebral dysfunction. Surface electromyography was performed on the right wrist flexor and extensor muscles, and negative myoclonus was recorded, characterized by a 300-milliseconds burst and a silent period when the wrist was outstretched. Initially, the patient was diagnosed with epileptic negative myoclonus and treated with 1000 mg of valproate sodium per day, which partly reduced the frequency of the symptoms. Two months after discharge, the patient was admitted due to altered mental status, dyspnea, and severe myoclonus. The follow-up EEG did not show any epileptiform discharges and exhibited only slowing. Arterial blood gas demonstrated severe hypercapnia (pO_2_ 44.1 mmHg, pCO_2_ 79.5 mmHg). Other laboratory tests of hepatic and renal functions were within normal limits. Valproic acid was discontinued, and the patient was intubated. Fifteen days after discharge, the negative myoclonus had completely disappeared. This case illustrates the importance of considering asterixis associated with respiratory failure in the differential diagnosis for the timely management of these individuals [118].

### 5.8. Electrolytes Abnormalities and Asterixis

Hypokalemia has been associated with asterixis and is also commonly seen in patients with alcoholic cirrhosis. Conn et al. reported asterixis in a cirrhotic patient with mild hypokalemia. According to the authors, the patient did not present any condition or laboratory abnormality that justified the depletion of these substances other than cirrhosis [117].

Asterixis was already associated with decreased [119] and increased [120] magnesium serum levels. Decreased levels of magnesium are commonly observed in individuals with alcoholic hepatitis, so the cases presenting hypomagnesemia associated with asterixis may be only anecdotal [119]. Morimatsu et al. described a case of chronic magnesium laxative use presenting with asterixis, in which the severe asterixis of the whole body gradually decreased in severity along with the decreasing magnesium concentration [120]. Also, hypermagnesemia, when associated with hypocalcemia, may induce choreiform movements and seizures [121].

Asterixis was already reported as the presenting symptom of hypercalcemia secondary to the parathyroid hormone-related protein in the setting of prostatic adenocarcinoma [122]. Also, there are cases of asterixis with hypercalcemia due to primary hyperparathyroidism [7]. An important differential diagnosis, especially in African Americans, is sarcoidosis in individuals presenting with asterixis because of the association of sarcoidosis with hypercalcemia and liver dysfunction [123].

Albumin levels are markedly correlated with liver dysfunction and are used in many medical calculators regarding liver transplant and prognosis. However, Pal et al. did not find any correlation between albumin levels and the severity of asterixis [7].

Hypophosphatemia has also been reportedly associated with encephalopathy and asterixis [124]. In a case series of twenty-six patients with a syndrome of inappropriate secretion of antidiuretic hormone, neurological signs were present in 18 of the 26 patients. They were regularly seen in patients whose serum sodium concentration was reduced to 115 mEq/L or less. Asterixis was observed in one of these patients with hyponatremia [125].

### 5.9. Other Causes of Asterixis

Among the causes of asterixis, episodic ataxias were already associated with asterixis. Lee et al. reported a case of episodic ataxia type 1 individuals presenting with asterixis, in which a novel KCNA1 mutation was observed [126]. This case expanded the genetic and clinical spectrum of episodic ataxias and can suggest the involvement of the KCNA1 mutation in the development of asterixis. KCNA1 channels were specifically found in the hippocampus, thalamus, neocortex, and ventral brain cortex, including the piriform and entorhinal cortex and the amygdala in mouse models [127].

Asterixis is commonly associated with thalamic lesions. Mears et al. reported a high-frequency focused ultrasound thalamotomy to manage essential tremors. The patient received seven sonications in the right thalamic regions. Soon after the procedure, he developed asterixis involving the left shoulder and leg, which progressively improved within six months [128]. This report is important because it supports the idea of asterixis as a manifestation of intermittent failure in maintaining sustained muscle contraction and tonic posture. Furthermore, it can support the thalamic involvement in the development of asterixis [128].

Asterixis is usually reported with central lesions. To be more specific, the most common reports are above the level of pons. But it can be rarely associated with peripheral lesions. In this way, peripheral presentations are probably pseudo-asterixis instead of true asterixis. Maramattom et al. reported an acute demyelinating neuropathy with asymmetric lower limb proprioceptive involvement, which impaired the afferent loop and caused peripheral asterixis [129]. One possible explanation for the development of the electrical findings could be abnormal reinnervation of the peripheral motor system, which can be supported by quick improvement within a week.

Umemura et al. reported a patient with progressive non-fluent aphasia who suffered from rhythmic myoclonus and asterixis [130]. During myoclonus episodes, focal hyperperfusion in the bilateral precentral gyri, which did not disappear in the absence of jerks, was observed [130]. This finding suggests that persistent hyperactivity in the selective prefrontal region can lead to myoclonus. Also, the focal hyperactivity lasted too long to be considered a result of myoclonus, though hyperperfusion can last several days due to anaerobically glycolytic metabolites in abnormal electrical activity [131]. Interestingly, this case can support the hypothesis that the stimulation of a specific frontal cortical area is related to asterixis [7].

Poersch et al. reported the case of a 58-year-old man with chronic paranoid-hallucinatory psychosis who presented with drug-induced asterixis (clozapine, benperidol) worsened by relative hypoglycemia [132]. The asterixis disappeared after oral antidiabetics were reduced, indicating that hypoglycemia might precipitate asterixis [132]. Also, this report can suggest the involvement of the decreased neuronal metabolism due to hypoglycemia leading to a hyper-excitatory state and overproduction of glutamine in a similar mechanism as proposed for hepatic encephalopathy.

## 6. Management

A clinician should remember that the evaluation and management of asterixis depends on the underlying disease process, and one should consider a wider differential diagnosis based on clinical history. The treatment of asterixis is the treatment of the underlying pathology. 

It is noteworthy that valproic acid, phenytoin, and carbamazepine, for example, could worsen asterixis secondarily to increased serum ammonia levels. Right after initiation of treatment, it is possible that a mild improvement is seen in symptoms, such as in the case reported by Kim et al. of a patient with asterixis in the context of respiratory failure who was first given valproic acid with a mild initial improvement and severe worsening of the symptoms two weeks later [118]. This initial improvement probably occurs due to the antiseizure effect of these medications. However, as the ammonia levels increase with time, asterixis may worsen. Therefore, these classes of medications should be cautiously used.

### 6.1. Asterixis in Hepatic Encephalopathy

Hepatic encephalopathy is defined as a spectrum of neuropsychiatric abnormalities in patients with liver dysfunction after the exclusion of brain disease [133]. A raised serum ammonia level is the classic laboratory abnormality reported in patients with hepatic encephalopathy. Triphasic and high-amplitude low-frequency waves are classic EEG changes associated with hepatic encephalopathy. Neuroimaging should be performed to rule out intracranial lesions when the diagnosis of hepatic encephalopathy is in question [134].

Because of the increased dietary fiber content, a natural cathartic, and decreased levels of aromatic amino acids, diets containing vegetable proteins appear to be better tolerated than diets rich in animal proteins, especially proteins derived from red meats. Aromatic amino acids are precursors of the false neurotransmitters, tyramine and octopamine, which are thought to inhibit dopaminergic neurotransmission and worsen hepatic encephalopathy. Lactulose appears to inhibit intestinal ammonia production by several mechanisms. It produces acidification of the gut lumen by conversion of lactulose to lactic acid and acetic acid [135]. This enhances the conversion of ammonia (NH3) to ammonium (NH4+); owing to the resultant relative impermeability of the membrane, the NH4+ ions are not easily absorbed and hence get trapped in the colonic lumen and reduce the plasma NH3. Gut acidification inhibits the ammoniagenic coliform bacteria, which leads to increased levels of non-ammoniagenic lactobacilli. Patients should take sufficient lactulose to have two to four loose stools daily. 

In 2013, a meta-analysis confirmed the utility of lactulose in managing hepatic encephalopathy [136]. Multiple clinical trials have demonstrated that rifaximin at a dose of 400 mg taken orally three times a day was as effective as lactulose or lactitol at improving hepatic encephalopathy symptoms [137]. A potential mechanism for rifaximin’s clinical activity is its effects on the metabolic function of the gut microbiota rather than a change in the relative bacterial abundance. The approval of rifaximin was based on a phase 3 clinical trial conducted by Bass et al. L-ornithine L-aspartate (LOLA) is a stable salt of the two constituent amino acids. L-ornithine stimulates the urea cycle, resulting in ammonia loss [138]. Both L-ornithine and L-aspartate are substrates for glutamate transaminase. LOLA was found to be effective in treating hepatic encephalopathy in several European trials [139].

### 6.2. Uremic Encephalopathy

Patients with uremic encephalopathy may present with stimulus-sensitive myoclonus, asterixis, or both, which generally improve after dialysis or renal transplantation [140]. Most patients with chronic renal disease will not have complaints, but their detailed neurological examination will show asterixis. Also, the chronically elevated creatinine and urea levels in individuals undergoing dialysis are probably associated with the development of asterixis, and sometimes, even with an optimal dialytic regimen, asterixis will not improve.

### 6.3. Genetic Etiologies and Deep Brain Stimulation

Miyata et al. reported an individual presenting with asterixis and dystonia with a KMT2B mutation. His neurological symptoms were severe, and deep brain stimulation of the globus pallidus was performed, showing a significant improvement in dystonia and asterixis. Improving both movement disorders can suggest a common pathologic mechanism for these two pathologic movements [141]. The globus pallidus stimulation and significant asterixis improvement suggest that the postural stability and tonic control may be partially modulated by the globus pallidus [142]. Interestingly, thalamic lesions can lead to the development of asterixis, so patients undergoing thalamic targets for the management of refractory neurological conditions should be cautiously assessed in the follow-up for asterixis because this abnormal movement can affect the procedure’s efficacy.

### 6.4. Others

In cases of Wilson’s disease, the diagnosis is made when the serum ceruloplasmin seems to be low, and the copper concentrations result in high urinary copper levels, when there are suggestive brain MRI abnormalities, and Kayser–Fleisher rings where there are copper depositions in the periphery of the cornea seen during the slit lamp examination. The brain MRI features are diffuse and symmetrical T2-weighted hyperintensities in the striatum, thalamus, brainstem, cerebellum, and possibly white matter. Treatment consists of a low copper diet and copper chelating agents such as D-penicillamine and trientine or zinc, which interfere with the absorption of copper. Liver transplantation could be considered a rescue option in patients with severe neurological forms (albeit without severe necrotic lesions) that are resistant to anti-copper therapies [143].

## 7. Limitations, Recommendations and Future Studies

There are some limitations in the present study. Most importantly, this is not a systematic review. The lack of randomized clinical trials and large prospective cohorts about this condition prompted us to include case reports and case series in order to review the literature thoroughly. However, the incidence of this condition could be even higher than is documented. Future studies should assess the causes of asterixis in pediatric individuals. There is a significantly lower number of studies regarding this specific part of the population. One of the factors associated with the scarce discussion in the literature could be the overlap between asterixis and other movement disorders in these individuals, leading to delayed diagnosis or even misdiagnosis of the abnormal movement. 

We have observed that most studies regarding non-hepatic causes of asterixis were written in the 60s. This could be explained by better physical exam skills and more time dedicated to observation of the patients among internal medicine specialists at that time. In this way, researchers should also review the pathophysiological mechanisms underlying non-hepatic causes of asterixis, which is probably more prevalent than is currently known. 

The development of new drugs will probably be associated with the occurrence of asterixis, especially on two occasions. First, drug classes already reported with asterixis, which can be explained by class effect and similar interaction with receptors. Second, drugs or classes of drugs that cause encephalopathy will probably be associated with asterixis.

Another subject that should be further studied is the epileptic negative myoclonus. Most studies are from the 1990s, and no specific description of the pathophysiology is provided. The classification was based on electrodiagnostic studies, but specific experiments with this group of conditions were not done.

Asterixis is one of the most well-known physical findings of the neurological examination in hepatic encephalopathy. An important historical factor related to this is the presence of specific chapters in some older versions of Harrison’s Principles of Internal Medicine, as described by Pal et al. [7]. We highlighted other important facts related to asterixis in a table (Table 7).

## 8. Conclusions

In sum, asterixis is usually asymptomatic and not spontaneously reported by patients. It should be actively searched for in the physical exam of encephalopathic patients and when present, it should raise suspicion for an underlying toxic or metabolic cause. It is not specific to any pathophysiological process, but it is more commonly reported in hepatic encephalopathy, renal and respiratory failure, cerebrovascular diseases, as well as associated with drugs that could potentially lead to hyperammonemia, such as valproic acid, carbamazepine and phenytoin. Asterixis is usually reversible upon treatment of the underlying cause. Thus, asterixis is a highly unspecific finding, especially for diagnosing any systemic disorder at an early stage. Stroke is the most common cause of unilateral asterixis, occurring on the side contralateral to the lesion.

## Figures and Tables

**Table 1:** Positions for examination of asterixis proposed by Pal et al. [7].

1. Arms extended and pronated with wrist extension for 10 s
2. Arms extended and pronated without wrist extension for 10 s
3. Arms extended and supinated without wrist extension for 10 s
4. Adduction of arms with partial flexion of elbows for 5 s
5. Straight leg raising with maximal dorsiflexion of feet for 5 s. Patients unable to raise their legs were tested with hips and knees flexed and feet dorsiflexed.
6. Straight leg raising without maximal dorsiflexion of feet for 5 s. If the patients could not raise their legs, they were tested with hips and knees flexed without dorsiflexion of the feet.
7. For patients unable to cooperate for lower extremity testing, the examiner would hold the feet together after setting the legs up in a hip flexion–knee flexion position to watch for flapping abduction–adduction movements of the legs.

**Table 2:** Asterixis classification based on the severity proposed by Pal et al. [7].

Graduation	Severity	Description
I	Minimal	Finger joints affected
II	Mild	Finger affected, slight movement of wrist or ankles
III	Moderate	Fingers affected, substantial movement of wrist and ankles, proximal joints slightly affected
IV	Severe	Moderate asterixis with substantial movement of the face or proximal extremities

**Table 3:** Bilateral asterixis secondary to focal brain lesions.

Reference	Age, Sex	Etiology	Confounding Factors	Lesion Site	Cause of Bilaterality
Bril et al. [29]	71, M	Infarction	None	Rostral midbrain tegmentum	Midline lesion
Weinreb et al. [30]	67, F	Hemorrhage	Phenytoin, cimetidine	Frontal hemorrhage	Parasaggital lesion and hydrocephalus
Santamaría Cano et al. [31]	NA	Subdural hematoma	None	Unilateral large subdural hematoma with compression of lateral ventricles	Midline shift
Guberman et al.-Case 1 [32]	54, M	Infarctions	Hypoxia	Bilateral medial thalami	Bilateral lesions
Noda et al. [33]	42, F	Tumor	None	Frontal lobe neuroblastoma with compression of the lateral ventricle	Midline shift
González et al. [34]	NA	Subdural hematoma	Hepatic disease	Bilateral frontal subdural hematomas	Bilateral lesions
Palomo et al. [35]	NA	Subdural hematoma	None	Bilateral subdural hematomas	Bilateral lesions, bilateral mass effect on lateral ventricles
Velasco et al.-Case 4 [36]	NA	Metastases	None	Bilateral thalamic/basal ganglia lesions	Bilateral lesions
Nokura et al. [37]	66, F	Tumor	None	Hypothalamus, thalamus, midbrain bilaterally	Bilateral lesions
Murakami et al. [38]	83, F	Infarction	None	Bilateral frontal lobe	Bilateral frontal lobes perfused by the anterior cerebral artery

Abbreviations: F, female; M, male; NA, not available/not reported.

**Table 4:** Unilateral/asymmetric asterixis associated with contralateral brain lesion.

Location	Reference
Subdural hematoma and frontoparietal lobe	[20,39]
Prefrontal area and cingulate gyrus	[40]
Corona radiata	[31]
Thalamus-corona radiata	[41]
Thalamus	[42]
Thalamus-midbrain	[43]
Thalamus-internal capsule	[44]
Basal ganglia-internal capsule	[45]
Internal capsule	[46]
Internal capsule (posterior limb)	[47]
External capsule	[45]
Putamen	[48]
Lenticular nucleus	[49]
Anterior centrum semiovale	[7]
Mibrain	[50]
Midbrain-cerebral peduncle	[51]
Watershed brain area	[52]
Cerebellum	[53]

**Table 5:** Classification of hepatic encephalopathy (the West Haven criteria) based on Basile et al. [59].

Grade	Level of Consciousness & Behavioral Changes	Neuromuscular Exam Findings
0	Normal	Normal
1	Mild confusion, shortened attention span, mild lack of awareness	Impaired addition or subtraction, mild incoordination, and difficulty with handwriting
2	Disorientation, personality changes, lethargy, inappropriate behavior	Asterixis, paratonia, apraxia, dysarthria
3	Somnolent but arousable, amnesia, rage, bizarre behavior, severe disorientation	Hyperreflexia, Babinski’s sign, spasticity
4	Coma	Thalamic–subcortical spasticity of the entire body; no response to painful stimuli

**Table 6:** Drug-induced asterixis.

Antibiotics: amphotericin B [7], cefuroxime [73], ceftazidime [73], cefepime [73], chloramphenicol [74], tretinoin [75], trimethoprim-sulfamethoxazole [76]
Anticholinergic: oxybutynin chloride [7]
Antiemetics: metoclopramide [77], promethazine [78]
Antiparkinsonian: amantadine [79], levodopa [80]
Antipsychotics: clozapine [70], olanzapine [7], risperidone [7]
Antiseizure medications: bromide [7], carbamazepine [81], gabapentin [82], lamotrigine [83], phenobarbital [84], phenytoin [85], pregabalin [86], primidone [84], valproate [87]
Benzodiazepines: clonazepam [7], lorazepam [7], metrizamide [88]
Histamine receptor modulators: famotidine [7]
Mood stabilizers: lithium [89]
Opioids: hydromorphone [7], meperidine [90]
Others: ammonium chloride [91], bumetanide [92], ifosfamide [93], iopamidol [94], salicylates [95]

**Table 7:** Clinical clues in asterixis.

1. Asterixis usually presents with bilateral motor symptoms and is commonly associated with encephalopathy secondary to metabolic abnormalities or the toxic effect of some medications. However, asterixis is a non-specific sign of cerebral pathology.
2. Asterixis is an asynchronous pathology, except when involving facial muscles.
3. Unilateral asterixis is most commonly associated with contralateral brain lesions.
4. In bilateral asterixis, where metabolic and drug causes were ruled out, vascular lesions should be investigated, especially in the midbrain-thalamic region.
5. Asterixis alone can help guide clinicians regarding indications for admission.
6. Patients with asterixis are usually asymptomatic, and their main complaints are related to recurrent falls and abnormal handwriting. Some patients with near falling complain of “weak legs” or legs “giving out.” Others complain of jerky handwriting, inability to write with one hand on a pad held up with the other, or difficulty typing.
7. Asterixis is a reversible movement disorder. Treating the primary cause can lead to full recovery of the jerks.
8. Asterixis is more easily observed in patients with previous disabilities such as blindness. This can be explained by the correctional movements related to other symptoms including the cerebellum and visual system.
9. Trunchal and proximal asterixis could affect the deltoid and hip flexor muscles. In these cases, the individuals can be misdiagnosed as functional, especially in patients with neuropsychological comorbidities.
10. Asterixis should be investigated in patients with confusion or impaired alertness.

## Data Availability

Not applicable.
